# The Effect of Performing a Dual Task on Postural Control in Children with Autism

**DOI:** 10.1155/2013/796174

**Published:** 2013-10-30

**Authors:** Maria Pia Bucci, Catherine Doyen, Yves Contenjean, Kelley Kaye

**Affiliations:** ^1^UMR 676 Inserm - Université Paris Diderot, Hôpital Robert Debré, 48 Bd Sérurier, 75019 Paris, France; ^2^C.R.E.D.A.T. Centre de Recherche et de Diagnostic de l'Austime et des Troubles Apparentés, Centre Hospitalier Sainte Anne, 1 rue Cabanis, 75674 Paris, Cédex 14, France; ^3^Laboratoire de Psychologie et Processus de Santé, LPPS EA 4057, Université Paris Descartes, 92774 Boulogne-Billancourt Cedex, France

## Abstract

The aim of the study was to explore the effect of eye movements (saccades and pursuits) on postural stability in children with autism versus typically developing children of comparable age. Postural stability was recorded with a platform (Techno Concept) in seven children with autism (mean age: 6 ± 0.8) while fixating a target or making saccades or pursuit eye movements. Data was compared to that of seven age-matched typically developing children. Surface area and mean speed of the center of pressure (CoP) were measured. Autistic children (AC) were more instable than typically developing children (TD), both in simple as well as dual task conditions. Performing a dual task thus affects AC and TD children in a different way. AC stability is not improved during saccades or pursuit eye movements in the dual task condition; in contrast, saccades significantly improve postural stability in TD children. The postural instability observed in AC during simple as well as dual task supports the hypothesis that such children have deficits in cerebellar functions.

## 1. Introduction

Autism is a common neurodevelopmental disorder that is diagnosed on the basis of anomalies in social interaction, verbal and nonverbal communication, and cognitive flexibility that are apparent before the age of three [1]. It has been noted that between 25% and 50% of diagnosed individuals never acquire functional language [2, 3]. Although not currently included as diagnostic criteria for autism, processing abnormalities across sensory domains are often observed [4] and are included in the Childhood Autism Rating Scale [5] and the Autism Diagnostic Interview-Revised [6] (ADI-R).

Abnormalities of motor coordination, posture, and gait are also frequently associated with autism [7].

Postural control observed in children with autism appears to differ from that of typically developing children (TD). Indeed, Gepner et al. [8] and Gepner and Mestre [9] showed that autistic children are more unstable than control children of comparable age; they suggested that the instability observed in these kinds of children could be due to cerebellar deficits responsible for regulation of visual, vestibular, and somatosensory information. Abnormal sensory input integration for good postural control in autistic children has also been reported by Molloy et al. [10] when visual and/or somatosensory inputs were modified or eliminated during postural recording. Gowen and Miall [11] and Rinehart et al. [12] also suggested that a deficit at the basal ganglia and cerebellum level could be at the origin of postural instability in children as well as adult subjects with autism.

Postural control has been considered to be an automatic system [13], but recent studies suggested that attentional processes are involved in the regulation of posture during simple or more complex tasks, especially when the latter involves attentional processes [14, 15].

In children, it is well known that visual information plays an important role for postural control [16, 17]; visual inputs are known to require attention, and attention is also involved in the execution of eye movements [18]. Several structures of the central nervous system in the cerebral cortex (frontal, parietal, and occipital) and in the brainstem (paramedian pontine reticular formation and superior colliculus) play an important role in postural control as well as in the programming and execution of eye movements [19]. Consequently, one could expect interferences between oculomotor and postural control. Studies on the effects of eye movements on posture in normal children are inexistant. Few studies have focused only on adult subjects, and their results are discordant.

White et al. [20] compared postural stability during a fixation and a saccadic task, and they did not find any change in postural parameters in these two visual conditions. In contrast, others studies [21–23] reported an improvement in postural stability during saccadic eye movements. Also, Strupp et al. [24] observed that pursuit eye movements led to an impairment in postural stability in normal adult subjects. Recall that making eye movements requires an active state of attention to fixate (saccades) or to follow (pursuits) the target; in other words, studying the effect of eye movements on postural control could be a useful tool to examine the interaction between eye movements, visual attention, and body stability.

The aim of the present study was to explore further the extent of the effect of eye movements (saccades and pursuits) on postural control in children with autism versus typical developing children of comparable age.

## 2. Material and Methods

### 2.1. Platform Posturography

A platform which functions according to the principle of strain gauge and consists of two dynamometric clogs (Standards by Association Française de Posturologie, produced by TechnoConcept, Céreste, France) was used to measure postural stability. This platform is a normalized AFP40/16 Stabilotest. The excursions of the center of pressure (CoP) were measured for 12.8 sec, and the surface of the CoP was calculated following Gagey's standards [25]; the equipment included a 16-bit analogue-digital converter, and the acquisition frequency was 40 Hz.

### 2.2. Participants

Seven children (5 boys and 2 girls, ranging in age from 3 to 8 years old; mean 6 ± 0.8) who had received a diagnosis of childhood autism according to ICD-10 diagnostic criteria participated in this study. Diagnostic procedures included extensive testing by a multidisciplinary team of professionals who are specialized in pervasive developmental disorders and who work together in an accredited diagnostic and research center in Paris, France. Each diagnosis of childhood autism was confirmed by means of scores obtained on the Autism Diagnostic Interview-Revised (ADI-R6), and the intensity of autistic symptoms was assessed by means of the Childhood Autism Rating Scale [5]. The Psychoeducational Profile-Revised Schopler et al. [26] was used in order to assess each child's global developmental age and calculate his or her global developmental quotient. All subjects presented with significant developmental delay (mean QD = 40, range = 24 to 65) and had little or no expressive language. The global intensity of autistic symptoms ranged from mild to severe (mean global CARS score = 37, range = 31 to 41).

Seven age- and gender-matched typically developing (TD) children (5 boys and 2 girls, mean age: 6 ± 17) were also tested. 

The investigation adhered to the principles of the Declaration of Helsinski and was approved by our institutional Human Committee. Informed parental consent was obtained for each subject after the nature of the procedure had been explained.

### 2.3. Visual Tasks

Three visual tasks were designed: fixation, saccades, and pursuit tasks. Stimuli were presented on the PC screen (19 inches, resolution: 800 × 600 pixels, and luminance of background: 7 cd/m^2^) adjusted at the eye level in front of the child. It should be noted that eye movements are not recorded in this study.

#### 2.3.1. Fixation Task

The fixation target was a smiley face (1.4°), and it was displayed at the center of the white screen during all the time of postural recording (12.8 sec). The child was invited to fixate the smile.

#### 2.3.2. Saccadic Task

Visually guided saccades were elicited by using a simultaneous paradigm to induce reflexive saccades. At the start of each trial, a central black square of 1.4° was switched on for a period of 1500 ms; afterwards the square was switched off, and simultaneously a target (little green man, smiley) measuring 1.4° appeared at the periphery of the screen (eccentricity of the target was 10° to the right or left, up or down) and stayed on for 1500 ms. Children were invited to make horizontal or vertical saccade to the target. A total of 9 saccades were simulated for the postural recording.

#### 2.3.3. Pursuit Task

The target was a dot (1.4°) moving horizontally across the PC screen at 0.2 Hz. Subjects were invited to follow the target visually.

For each visual task condition, two trials were recorded for each child (each lasting 12.8 sec). The order of the visual tasks varied randomly across children. Children were asked to stay as stable as possible, with the arms along the body.

### 2.4. Postural Recording Procedure

Each child stood independently on the platform, in front of the screen located 60 cm away from him at eye level. For each visual task two postural recordings were taken successively. Subjects were asked to stay as stable as possible, with the arms along the body. In order to be sure that instructions were correctly understood, by both verbal and nonverbal subjects, target behaviors were modeled by the experimenter (stepping onto the platform, keeping still with arms along the body, and paying attention to the stimuli on the screen), and children were trained to dual task before starting postural recordings.

### 2.5. Data Analysis

To quantify the effect of visual tasks on the postural performance we analyzed the surface area and the mean speed of the center of pressure (CoP). The surface area allowed the measurement of the CoP spatial variability [27], and the mean of speed represents a good index of the amount of neuromuscular activity required to regulate postural control [28].

Statistical analysis was performed by the Mann-Whitney *U* test to compare the two groups of children in the different dual task conditions; furthermore, for each group of children tested, the Friedman test was also run for comparison between the three different conditions. The effect was significant when the *P* value was below 0.05.

## 3. Results

**Figure 1:**
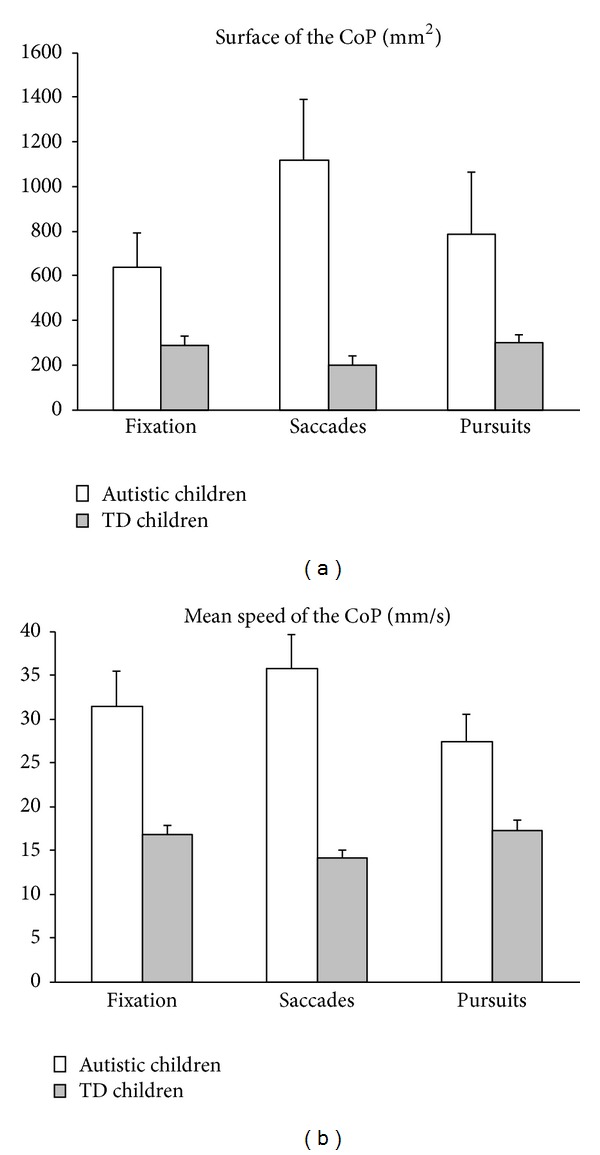
Mean of surface area (a) and mean speed of CoP (b) in children with autism (AC) and in typically developing children (TD), during fixation, saccades, and pursuit eye movements. Vertical bars indicate the standard error.

Figure 1 shows the postural parameters (surface area and mean speed of the CoP) that were measured during the three experimental conditions (fixation, saccades and pursuit eye movements) for autistic and typically developing children. Concerning the surface of the CoP (Figure 1(a)), the Mann-Whitney *U* test showed a significant larger value of the surface area of the CoP for autistic children with respect to typically developing children for each of the three visual conditions tested (*Z* = 1.98, *P* < 0.04 during fixation task; *Z* = 3.13, *P* < 0.001 during saccades task; and *Z* = 2.49, *P* < 0.01 during pursuit task).

Figure 1(b) shows the data obtained concerning the mean speed of the CoP. The Mann-Whitney *U* test showed a significant larger value of the mean speed of the CoP for autistic children with respect to typically developed children for each of the three visual conditions tested (*Z* = 2.61, *P* < 0.009; *Z* = 3.13, *P* < 0.001; and *Z* = 2.10, *P* < 0.03, resp., for the fixation, saccades, and pursuit task).

Let us now examine the dual task, which is the effect of eye movements on postural stability. The dual task was different for the two groups of children examined. For autistic children the Friedman test showed for the surface area of the CoP a significant difference between the three visual conditions (*χ*² = 7.7, *P* < 0.02). The smaller surface area of the CoP was observed for the fixation task (638 ± 150 mm^2^) and the larger value during the saccades task (1115 ± 271 mm^2^); pursuit eye movements led to intermediate values of the surface area of the CoP (784 ± 277 mm^2^). The Friedman test failed to show any significant difference in the three different conditions for the mean speed values of the CoP (*χ*² = 2.5, *P* = 0.2); indeed, values were similar during fixation (31 ± 4 mm/s), the saccades task (35 ± 3.8 mm/s), and pursuits (27 ± 3 mm/s).

In contrast, typically developing children's behavior was different because eye movements affected both the surface area and the mean speed of the CoP. A Friedman test showed a significant difference of the surface area of the CoP for the three visual conditions (*χ*² = 10.6, *P* < 0.005). The smaller surface area of the CoP was observed in the saccades task (200 ± 39 mm^2^) and the larger value during the pursuit task (300 ± 32 mm^2^); the fixation task led to intermediate values of the surface area of the CoP (286 ± 43 mm^2^). 

The Friedman test also showed a significant difference of the mean speed of the CoP for the three visual conditions (*χ*² = 10.57, *P* < 0.005). The smaller value of the mean speed of the CoP was observed in the saccades task (14 ± 0.9 mm/s) and the larger value during pursuit task (17 ± 1 mm/s); the fixation task led to intermediate values of the mean speed of the CoP (16 ± 1 mm/s).

## 4. Discussion

The main findings from this study are as follows: (i) children with autism are more unstable than typically developing, age-matched children both in simple as well as dual-task conditions; (ii) dual-task affects typically developing and autistic children in a different way. These findings are discussed next.

### 4.1. Poor Postural Control in Autistic Children

The present results confirm and enlarge findings already reported regarding the quality of postural stability in children with autism. Indeed, as exposed in the Introduction, several studies showed poor postural stability in autistic children suggesting deficiencies in the basal ganglia and cerebellum [11, 12]. Studies examining posture in children with cerebellar deficits [29, 30] have reported poor postural stability, suggesting that these children have difficulties integrating multimodal sensory information for balance control and/or in properly compensating the deficit of sensory input [31]. Based on our findings, we could assume that AC are not able to use sensorial input in order to assure good postural control. 

### 4.2. Different Effect of Saccades and Pursuits on Postural Control in Typically Developed Children

As mentioned in the Introduction, in healthy adult subjects the results relating to the effect of visual tasks on postural control are still controversial. Our results showed for the first time that postural stability in typically developing children is improved when the child is making saccades rather than fixating a target. During the saccadic task, the surface area of CoP decreased with respect to the fixation task. This result is in line with findings from other researchers [21–23, 32] which reported an improvement in the postural stability during saccadic eye movements in adult subjects. These authors attributed this effect to signals related to the initiation or/and execution of saccades, which are integrated by the postural system via vestibulospinal and reticulospinal signals in the muscle tone of the lower limb. Furthermore, Rougier and Garin [32] underlined the importance of attentional demands in postural control. Executing a secondary task (saccades) while performing a postural task allows attention not to be focused on postural control, which leads to better postural stability (automatic attentional system). Such improvement might be due to the fact that postural control becomes more automatic. 

Pursuit eye movements led to opposite results; typically developing children decreased their postural stability while performing these eye movements. This finding agrees with previous work from Strupp et al. [24] in normal adult subjects reporting that pursuit eye movements impaired postural stability.

Such diverging effects of different types of eye movements on postural control in children could be explained by the U-shaped nonlinear interaction model [33], which explored the effect of a secondary task on postural stability; the secondary task could decrease postural stability as a function of the attentional cost of such a task. For instance, the absence of a cognitive task (the simple fixation of a target) directs children's attention to postural control, thereby increasing the attentional resources needed to control posture; in contrast, a more complex task (i.e., pursuit eye movements) could be responsible for shifting the attention away from postural control, decreasing postural performance.

### 4.3. Dual-Task in Autistic Children

Independent of to the type of the secondary task (saccades or pursuits) performed during the postural task, autistic children decreased their postural stability. Indeed, the results show that the surface area of the CoP is smaller during the fixation task, and it increased during pursuits reaching larger values during saccades. This finding could be due to the poor oculomotor performances reported in these kinds of children [34, 35], but it could also be due to the fact that autistic children have difficulty in shifting attention [36]. Consequently, performing a dual-task is very difficult for them.

## 5. Conclusion

Postural control in autistic children is poor, in contrast to typically developing age-matched children, and their postural stability does not improve while performing a dual-task. This finding could provide some insight for adapting rehabilitation techniques to the needs of autistic children. For instance, the occupational therapist has to avoid dual-task conditions when aiming to improve postural control in these kinds of children. Finally, this finding must be replicated by studying a larger number of autistic children, and further investigation could focus on combining eye movement postural recordings techniques.
